# Amplicon sequencing of 42 nuclear loci supports directional gene flow between South Pacific populations of a hydrothermal vent limpet

**DOI:** 10.1002/ece3.5235

**Published:** 2019-05-06

**Authors:** Sophie Plouviez, Abigail Leavitt LaBella, David W. Weisrock, F. A. Bastiaan von Meijenfeldt, Bernard Ball, Joseph E. Neigel, Cindy L. Van Dover

**Affiliations:** ^1^ Department of Biology University of Louisiana at Lafayette Lafayette Louisiana; ^2^ Division of Marine Science and Conservation, Nicholas School of the Environment Duke University Beaufort North Carolina; ^3^ Department of Biology Vanderbilt University Nashville Tennessee; ^4^ Department of Biology University of Kentucky Lexington Kentucky; ^5^ Theoretical Biology and Bioinformatics Utrecht University Utrecht The Netherlands; ^6^ School of Biological, Earth & Environmental Sciences University College Cork Cork Ireland

**Keywords:** conservation genetics, deep‐sea, invertebrates, phylogeography, population genetics

## Abstract

In the past few decades, population genetics and phylogeographic studies have improved our knowledge of connectivity and population demography in marine environments. Studies of deep‐sea hydrothermal vent populations have identified barriers to gene flow, hybrid zones, and demographic events, such as historical population expansions and contractions. These deep‐sea studies, however, used few loci, which limit the amount of information they provided for coalescent analysis and thus our ability to confidently test complex population dynamics scenarios.

In this study, we investigated population structure, demographic history, and gene flow directionality among four Western Pacific hydrothermal vent populations of the vent limpet *Lepetodrilus* aff. *schrolli*. These vent sites are located in the Manus and Lau back‐arc basins, currently of great interest for deep‐sea mineral extraction. A total of 42 loci were sequenced from each individual using high‐throughput amplicon sequencing. Amplicon sequences were analyzed using both genetic variant clustering methods and evolutionary coalescent approaches. Like most previously investigated vent species in the South Pacific, *L*. aff. *schrolli* showed no genetic structure within basins but significant differentiation between basins. We inferred significant directional gene flow from Manus Basin to Lau Basin, with low to no gene flow in the opposite direction. This study is one of the very few marine population studies using >10 loci for coalescent analysis and serves as a guide for future marine population studies.

## 1 INTRODUCTION

Genetic homogeneity and panmixia were once assumed to be characteristic of marine species with planktonic larvae capable of dispersal over long distances (Scheltema, 1986). However, genetic variation surveyed from marine species revealed cases where the extent of population subdivision exceeded expectations based on the predicted larval dispersal (Burton & Feldman, 1982; Palumbi, 1994). Advances in next‐generation sequencing (NGS) technology have increased our ability to detect both population subdivision (Benestan et al., 2015; Hohenlohe et al., 2010) and, with adequate sampling, estimates of directional gene flow between marine populations.

Population subdivision can result from barriers to dispersal that include oceanic currents (Baums, Miller, & Hellberg, 2005; Thornhill, Mahon, Norenburg, & Halanych, 2008), geomorphology (Won, Young, Lutz, & Vrijenhoek, 2003), and vicariance events like the rising of the Isthmus of Panama (Bermingham & Lessios, 1993; Cunningham & Collins, 1994), or simply by persisting for long periods of time across a given area (Cunningham & Collins, 1998; Wright, 1951). At deep‐sea hydrothermal vents, comparative phylogeographic analyses of the mitochondrial cytochrome c oxidase subunit I gene (COI) have shown that tectonic history impacts population structure and demography (Hurtado, Lutz, & Vrijenhoek, 2004; Plouviez et al., 2009). The addition of nuclear loci and coalescent analysis (Johnson, Young, Jones, Waren, & Vrijenhoek, 2006; Plouviez, Le Guen, Lecompte, Lallier, & Jollivet, 2010) has further supported the importance of tectonic history on populations and provided additional resolution that has strengthened our understanding of hybrid zones and intergradation in vent‐endemic species (Faure, Schaeffer, & Fisher, 2015; Johnson, Won, Harvey, & Vrijenhoek, 2013; Plouviez et al., 2013; Zhang, Johnson, Flores, & Vrijenhoek, 2015). While these studies have provided important insight into gene flow and population dynamics, they were unable to determine the direction of migration due to the small number of loci (4–5) examined (Faure et al., 2015; Johnson et al., 2013; Plouviez et al., 2010). Due to the stochastic nature of the coalescent, single‐locus histories are often discordant and do not necessarily reflect population histories (Hare & Avise, 1998; Hey & Machado, 2003; Neigel & Avise, 1986; Palumbi & Baker, 1994). Increasing the number of individuals and loci sampled can improve inferences of population‐level parameters (Irwin, 2002; Maddison & Knowles, 2006).

We can now test more complex phylogeographic hypotheses and refine parameter estimates by increasing the depth, both in the number of individuals and the number of loci, of our genetic sequencing using individual tagging and high‐throughput amplicon sequencing (O'Neill et al., 2012). This approach can produce a large number of sequenced haplotypes (i.e., sequences for both alleles), which allows precise coalescent analysis of past and present population dynamics in hydrothermal vent populations.

**Figure 1:**
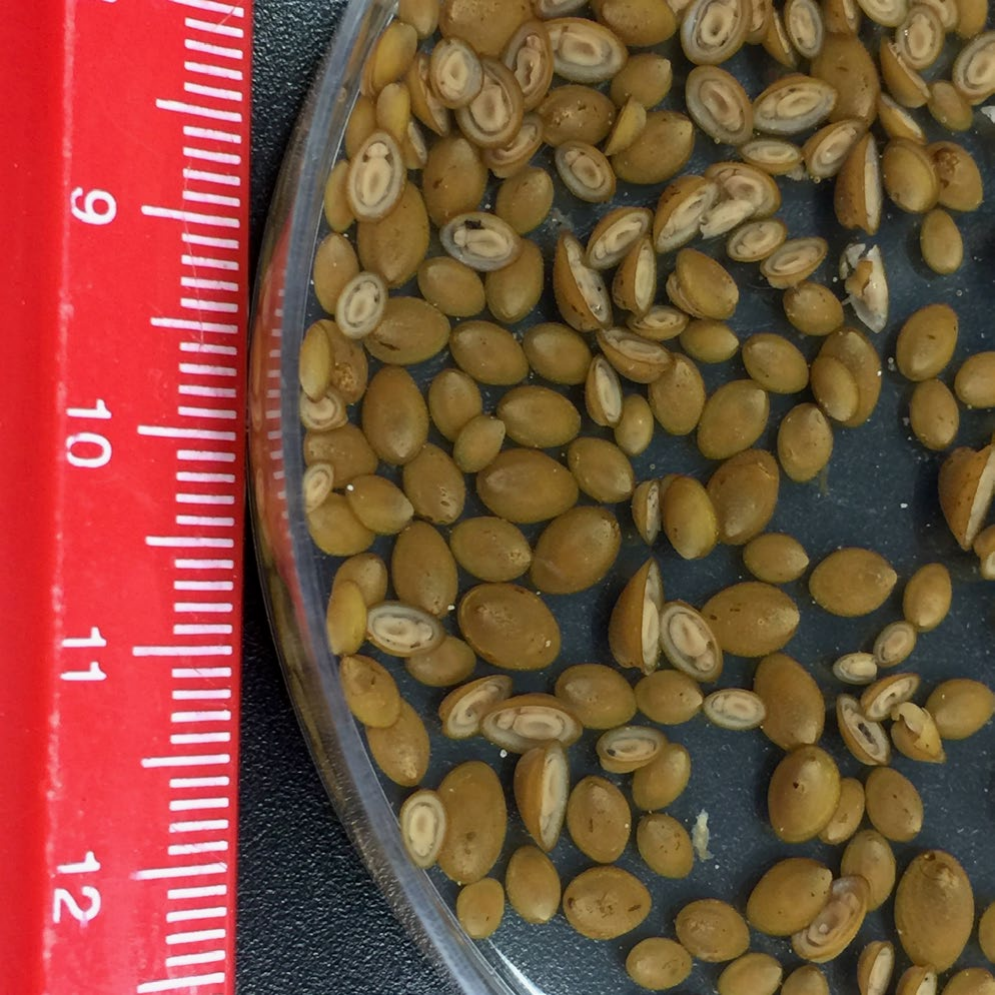
*Lepetodrilus* limpets collected from the Lau Basin and used in this analysis

**Figure 2:**
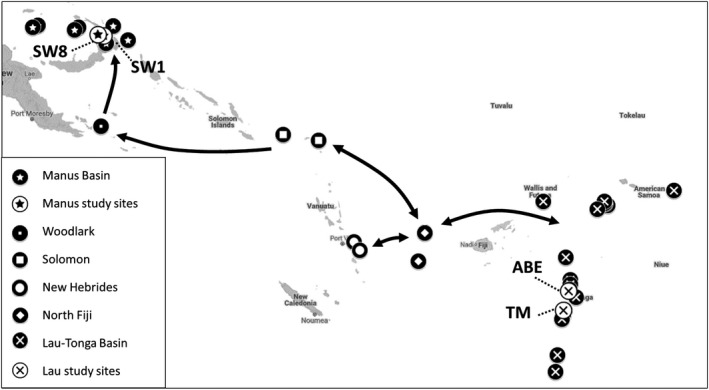
Verified hydrothermal vents in the South Pacific.Solwara 1 (SW1), Solwara 8 (SW8), Tu'i Malila (TM), and ABE were sampled for limpets. Arrows represent the direction of predicted larval transport between basins at 1,000 m with a PLD of 166 days (Mitarai et al., 2016)

In the Southwest Pacific, hydrothermal vents are home to a vast array of animals that are directly or indirectly supported by chemosynthesis, including *Lepetodrilus* limpets (Figure 1). These vents are distributed along multiple back‐arc basins (Figure 2). There is growing interest in mineral extraction at hydrothermal vents, especially in Manus (Papua New Guinea) and Lau (Tonga) Basins. Possible destruction of vents from mining necessitates an assessment of genetic variation and population connectivity to serve as baselines prior to extraction (Van Dover, 2011). Simulation studies of dispersal potential in the region have predicted high connectivity within most basins but varying connectivity between basins (Mitarai, Watanabe, Nakajima, Shchepetkin, & McWilliams, 2016; Suzuki, Yoshida, Watanabe, & Yamamoto, 2018). Between‐basin connectivity is predicted by Mitarai et al. (2016) to be predominantly northwest (Figure 2). Studies of genetic differentiation that included Manus and/or Lau Basins have revealed varying amounts of within‐ and between‐basin population structure (Table 1). Within basins, only one species showed population structure; *Munidopsis lauensis* (squat lobster) microsatellite data revealed significant differentiation between samples from Solwara 1 and samples from Solwara 8 and South Su sites (Thaler et al., 2014). In the other species studied (snails, shrimp, mussels, barnacles), no within‐basin population structure was detected in Manus, North Fiji, or Lau Basins (Kyuno et al., 2009; Plouviez et al., 2013; Suzuki et al., 2006; Thaler et al., 2014, 2011).

**Table 1:**
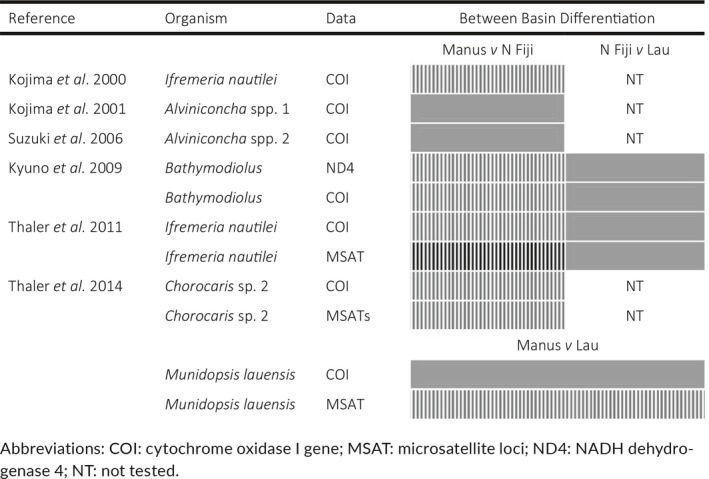
Population structure between Manus, North Fiji, and Lau basins. Striped boxes indicate population structure between two basins. Gray boxes indicate no detection of population structure between two basins

Genetic differentiation between Manus and North Fiji basins (Figure 2) has been detected using COI in three species, *Ifremeria nautilei* snails*, Bathymodiolus* mussel species, and *Chorocaris* sp. 2 shrimp (Kojima, Segawa, Fujiwara, Hashimoto, & Ohta, 2000; Kyuno et al., 2009; Thaler et al., 2014, 2011). In *I. nautilei* and *Chorocaris* sp. 2, genetic differentiation between Manus and North Fiji was confirmed with microsatellites (Thaler et al., 2014). Conversely, no population differentiation was found between Manus and North Fiji using mitochondrial sequences in two *Alviniconcha* species (spp. 1 and spp. 2) (Kojima et al., 2001; Suzuki et al., 2006). In *M. lauensis*, a recent selective sweep that reduced COI genetic diversity is suggested by one dominant haplotype across the Southwest Pacific (Thaler et al., 2014). Such a selective sweep would have removed any fingerprint of population differentiation for this mitochondrial gene, but population differentiation between Manus and Lau was detected using nuclear microsatellite markers (Thaler et al., 2014). An analysis based on model testing for migration following isolation in *I. nautilei* using COI suggested a lack of gene flow between Manus and North Fiji/Lau, but this analysis was based on only a single mitochondrial locus (Thaler et al., 2011).

To better understand population structure and demography of vent invertebrates in Manus and Lau, we investigated the limpet species *Lepetodrilus* aff *schrolli* (Figure 1; Johnson, Waren, & Vrijenhoek, 2008). The high abundance of *L*. aff. *schrolli* at active vents in the South Pacific allowed for collection of many individuals per site. Furthermore, COI analysis conducted in this study expands the known population range of *L*. aff. *schrolli* to include the Manus Basin. Previously, *L*. aff. *schrolli* was reported from the Fiji and Lau basins and *L. schrolli* was reported from the Manus Basin (Johnson et al., 2008). Unlike previous population studies in the region that used COI and microsatellite loci, we assessed the potential for genetic structure and restricted gene flow between basins using over 15 kb of aligned DNA sequences from 42 single‐copy intronic and exonic loci sampled from 93 individuals collected in the Manus and Lau Basins. Our extensive sequence data are more likely to meet the mutation models assumed in many coalescent‐based demographic inference programs that test models of isolation with migration (IMA2p software, Hey, 2010a; Hey, 2010b; Sethuraman & Hey, 2016). Furthermore, 33 of our 42 loci contain intronic sequences that are more variable and thus more likely to capture recent neutral variation which is suitable for analyzing population structure within a species or between closely related species (He & Haymer, 1997; Lessa, 1992; Palumbi & Baker, 1994). The use of a large multilocus dataset provided a sampling of gene genealogies that maximized our recovery of coalescent information representative of the genome. Thus, we were able to substantially expand on previous genetic work in this deep‐sea basin system.

Using both genetic population clustering and evolutionary coalescent approaches, we tested the hypothesis that *L*. aff. *schrolli* has been affected by tectonic history—through the creation of barriers or changes in distance among basins–similar to the pattern observed in other species: no genetic structure within basin, but genetic differentiation between Manus and Lau populations. We also tested the hypothesis that gene flow, if any, is consistent with larval dispersal models predicted in the region (Mitarai et al., 2016; Suzuki et al., 2018).

## 2 MATERIALS AND METHODS

### 2.1 Sampling

*Lepetodrilus* limpets were sampled at four hydrothermal vent sites (Figure 2; Table 2): two sites in Manus Basin (Solwara 1, Solwara 8) and two sites in Lau Basin (ABE, Tu'i Malila). *Lepetodrilus* limpets are known to graze bacteria on the shells of other vent invertebrates (e.g., *Ifremeria* sp. snails, *Bathymodiolus* sp. mussels) and rocks. Snails, mussels, and rocks were collected using the mechanical arm of remotely operated vehicles (ROVs, ST212 trenching ROV in Manus Basin and ROV Jason II in Lau Basin). Once aboard the ship, *Lepetodrilus* individuals were preserved at −80°C or in 95% EtOH for future nucleic acid extractions.

**Table 2:** Sample information for *Lepetodrilus* aff. *schrolli*

Sites	Basin	Latitude	Longitude	Depth (m)	*N*
Solwara 8 (SW8)	Manus	3.728°	151.681°E	1,720	23
Solwara 1 (SW1)	Manus	3.789°S	152.096°E	~1,490	24
ABE	Lau	20.763°S	176.191°E	~2,200	23
Tu'i Malila (TM)	Lau	21.989°S	176.568°E	1,880	23

### 2.2 Transcriptome and primer design

A transcriptome for *L*. aff. *schrolli* was generated as a reference to identify and sequence amplicon sequences from putatively single‐copy genes. RNA was extracted from two limpets from the Lau Basin using a Qiagen RNA Easy Plant Mini kit following the manufacturer protocol (Qiagen). Prior to RNA extraction, individuals were crushed together for ~15 s using a bead beater. Because of their small size, two individuals were combined to obtain 20 μg of RNA before ribosomal RNA depletion for transcriptome sequencing. Depletion (yield 200 ng of RNA), library preparation, and transcriptome sequencing on a Roche 454 GS‐FLX Titanium sequencer were performed by the Duke University Center for Genomic and Computational Biology.

Sequence reads were assembled de novo using the proprietary 454 assembler Newbler v2.6 (Margulies et al., 2005). The resulting contigs were then compared by reciprocal BLAST to the closely related *Lottia gigantia* genome (Grigoriev et al., 2012) to identify potential intron positions. Putative gene annotations were generated by comparing contigs to the *Drosophila melanogaster* genome using InParanoid7 (Ostlund et al., 2010). Primer pairs that flanked putative introns were designed using Primer 3 (Untergasser et al., 2012) with an annealing temperature within one or two degrees of 60°C. In addition to the primers designed to flank introns, primers to amplify the COI locus were included (Johnson et al., 2008). Nuclear primer pairs are detailed in Table S1.

### 2.3 DNA amplification, library construction, and sequencing

Genomic DNA from 23 to 24 individuals from each of the four sites (Table 2) was extracted using a cetyltrimethylammonium bromide (CTAB) method (Doyle & Dickson, 1987). Primer pairs were tested on two individuals. PCR amplifications were performed in 25 μl volume of: 1× MyTaq reaction buffer (Bioline), 2 mM of MgCl_2_, 0.05 mM of each dNTP, 0.48 μM of forward and reverse primers, 0.5 U MyTaq DNA polymerase (Bioline), 5 μl of DNA template, and sterile H2O. Thermocycler conditions were as follows: 94°C/2 min, 40 × (94°C/45 s, 60°C/1 min, 72°C/1 min), and 72°C/10 min. PCR amplification was visualized on agarose gels (1%) to test for successful amplification and for sizing of amplicons.

Primer pairs that yielded amplicons < 800 bp (2 × sequencing length of the 454 at the time of the study) from two test individuals were then deployed on the remaining 91 individuals (93 total individuals). Following PCR amplification, individuals were barcoded using the protocol described in O'Neill et al. (2012). Labeled amplicons were then pooled into two distinct libraries: amplified loci < 500 bp, and amplified loci of 500–800 bp. For the large amplicon library, each individual was sheared with a Bioruptor NGS at high intensity for 12 min at cycles of 30 s on/90 s off. Libraries were then sent to the Duke Center for Genomic and Computational Biology and sequenced on a quarter of a Roche 454 run each. Additionally, COI loci were PCR amplified, following the protocol of Johnson et al. (2008) and then Sanger sequenced for further species clarification. PCR products were purified using an Exonuclease 1/Antarctic Phosphatase Enzymatic Reaction (New England Biolabs). Big Dye Terminator (v3.1) chemistry followed by AMPure magnetic beads purification (Agencourt) was used to sequence amplicons in both directions on an ABI 3730xl DNA analyzer (Applied Biosystems International).

### 2.4 Allelic reconstruction

For each sequencing library, unassembled 454 reads were first sorted by individual barcode using an in‐house Perl script. The reads for each individual were trimmed, quality controlled, and sorted into different files by locus using SeqMan Ngen (DNASTAR). The resulting dataset contained one file for each successfully amplified locus for each individual. Amplicons in each file were phased independently using a “read‐only” phasing method (LaBella, 2017). In brief, the “read‐only” method constructs every possible allele within an individual by joining reads that have overlapping SNPs. This method was chosen to avoid parameter biases that can result from failure to capture rare alleles when using population‐based phasing methods (Garrick, Sunnucks, & Dyer, 2010; Lamina, Bongardt, Kuchenhoff, & Heid, 2008).

Sequencing or PCR amplification error can lead to reconstructing more than two alleles within an individual. Erroneous alleles generated due to sequencing errors were identified by extremely low sequencing coverage (less than 5% of the total reads) and subsequently removed. We were also able to remove alleles likely to be the result of PCR recombination (intra‐individual) by reconstructing recombination events at every SNP position between three putative alleles and removing the recombinant allele. For example, given three alleles for an individual (A1 = ABCD, A2 = abcd, and A3 = ABcd), the recombinant allele can be identified as A3 since it is the only possible recombinant of any pairing (A1 vs. A2, A2 vs. A3, and A1 vs. A3). One (homozygous) or two (heterozygous) different consensus sequences were inferred for each individual. Using these methods, all loci sequenced had only two alleles per individual, supporting the hypothesis that these loci are single copy. Homozygous individuals were assigned two of the same sequence. Sequences from all individuals were aligned in AliView 1.09 (Larsson, 2014) using Clustal W 2.0 (Larkin et al., 2007).

Sequence alignments were checked by eye in AliView 1.09 (Larsson, 2014), and regions of uncertainty (e.g., repeated nucleotides, indels) were excluded from subsequent analyses. Because the analytic methods we used do not allow for recombination, the species alignments were then analyzed using IMgc (Woerner, Cox, & Hammer, 2007). IMgc removed potential recombinant individuals, recombinant regions, and nucleotide sites with >2 alleles in all samples. All sites in the alignment then followed an infinite model of substitution. For each gene, the longest block of nonrecombinant aligned regions was kept for the population genetics analyses and reported to GenBank.

### 2.5 Species analysis

Species designations were assigned by analyzing the COI loci sequenced using Sanger and 454 sequencing. These sequences were aligned in AliView 1.09 (Larsson, 2014) using Clustal W 2.0 (Larkin et al., 2007) with *Lepetodrilus* sequences from Johnson et al. (2008) and trimmed to minimize missing data. OTU clustering was computed in Mothur version 1.41.3 using the opticlust method with the Matthews correlation coefficient metric and a cutoff of 0.20 (Schloss et al., 2009). Haplotype networks were generated and visualized using PopART version 1.7 (http://popart.otago.ac.nz) and the Median‐Joining network method (Bandelt, Forster, & Rohl, 1999).

### 2.6 Population structure analysis

The number of genetically distinct populations was assessed using Structure 2.3.4 (Hubisz, Falush, Stephens, & Pritchard, 2009; Pritchard, Stephens, & Donnelly, 2000). For each locus, sequences were coded as haplotype numbers and individuals were assigned a diploid genotype. Structure uses a Bayesian Markov Chain Monte Carlo (MCMC) clustering method to assign individuals into *K* genetic groups. This approach both estimates the number of genetically distinct groups in the dataset and identifies potential migrants/admixed individuals among these groups. One to four potential genetic groups (*K* = 1–4) were tested with three replicates each using the no‐admixture model, 100,000 iterations for the burn‐in, and 1,000,000 iterations of data collection. Each basin was also analyzed independently using the same parameters except testing for one to three potential groups (*K = *1–3). Similar sets of analyses were also performed using an admixture model. In the no‐admixture model, individuals are discretely from one population or another, while the admixture model allows for individuals to have mixed ancestry (Hubisz et al., 2009; Pritchard et al., 2000). The most likely *K* was determined using Δ*K* (Evanno, Regnaut, & Goudet, 2005) generated by CLUMPAK (Kopelman, Mayzel, Jakobsson, Rosenberg, & Mayrose, 2015).

Population structure was also examined using a principal components analysis (PCA). Sequences were converted into EIGENSTRAT format (one genotype file, one SNP file, and one individual file) using a custom Perl script. Each locus was assigned to a different “chromosome” to account for linkage equilibrium. The PCA was run using the smartpca module from EIGENSOFT v6.1.4 (Patterson, Price, & Reich, 2006; Price et al., 2006).

### 2.7 IMa2p analysis

A model of isolation with migration was tested between the two distinct genetic groups using IMa2p (Hey, 2010a, 2010b; Sethuraman & Hey, 2016). IMa2p is a parallel version of IMa2 that uses coalescent models to estimate several model parameters: (a) a splitting time parameter (*t*), (b) a population parameter for contemporary and ancestral populations (*θ*_Lau_, *θ*_Manus_ *θ*_Ancestral_), and (c) a migration parameter in each direction (*m*_Manus→Lau_, *m*_Lau→Manus_). Parameter values are first unconstrained and estimated using a MCMC‐mode run. Sampled genealogies from this MCMC‐mode (M‐mode) run are then used into a Load‐genealogies mode (L‐mode) run to compare estimated parameters with estimates from all 25 nested demographic models of constrained *t*, *θ*, and *m* (Table 3).

**Table 3:** Joint posterior estimates of population sizes (*θ*) and migration rates (*m*) for all possible 25‐nested models, and their associated joint probability [log(*p*)] and log‐likelihood ratio statistic (2LLR). Nonrejected model (#3) and original model are shown in gray

Model	Log(*p*)	*df*	2LLR	*θ*_Manus_	*θ*_Lau_	*θ*_Ancestral_	*m*_Lau > Manus_	*m*_Manus > Lau_
Unconstrained *θ* parameters								
1—unconstrained migration rates	3.649		–	8.4995	3.0956	0.6447	0.1681	0.6872
2—equal migration rates	1.493	1	4.312*	8.8544	3.2703	0.6885	0.3402	[0.3402]
*3—no migration from Lau to Manus*	*3.001*	*1*	*1.294^NS^*	*9.4764*	*3.0266*	*0.7161*	*[0.00000]*	*0.7333*
4—no migration from Manus to Lau	−333.5	1	674.3***	8.6682	3.1069	0.8352	0.4168	[0.00000]
5—no migration	−1,384	2	2,776***	8.0779	3.3201	0.7697	[0.00000]	[0.00000]
*θ*_Manus_ = *θ*_Lau_								
6—unconstrained migration rates	−116.6	1	240.6***	5.8095	[5.8095]	0.6609	0.2886	0.3455
7—equal migration rates	−117.6	2	242.5***	5.8095	[5.8095]	0.6609	0.3140	[0.3140]
8—no migration from Lau to Manus	−363.2	2	733.6***	5.6022	[5.6022]	0.7239	[0.00000]	0.9819
9—no migration from Manus to Lau	−697.9	2	1,403***	5.5013	[5.5013]	0.8156	0.4581	[0.00000]
10—no migration	−1,854	3	3,715***	5.5550	[5.5550]	0.7697	[0.00000]	[0.00000]
*θ*_Manus_ = *θ*_Ancestral_								
11—unconstrained migration rates	−1,011	1	2,029***	5.4378	3.0746	[5.4378]	0.2388	0.5806
12—equal migration rates	−1,026	2	2,060***	4.7744	3.4282	[4.7744]	0.3984	[0.3984]
13—no migration from Lau to Manus	−1,230	2	2,467***	5.9115	2.7526	[5.9115]	[0.00000]	0.9217
14—no migration from Manus to Lau	−1,827	2	3,662***	5.1125	3.8995	[5.1125]	0.4581	[0.00000]
15—no migration	−3,018	3	6,044***	5.4144	3.3201	[5.4144]	[0.00000]	[0.00000]
*θ*_Lau_ = *θ*_Ancestral_								
16—unconstrained migration rates	−231.0	1	469.4***	8.6770	1.9417	[1.9417]	0.1895	0.9866
17—equal migration rates	−326.2	2	659.8***	8.6770	1.9417	[1.9417]	0.4465	[0.4465]
18—no migration from Lau to Manus	−427.6	2	862.6***	8.0161	2.2484	[2.2484]	[0.00000]	0.9217
19—no migration from Manus to Lau	−965.5	2	1,938***	8.6682	2.3729	[2.3729]	0.4168	[0.00000]
20—no migration	−2,074	3	4,156***	9.6747	2.5210	[2.5210]	[0.00000]	[0.00000]
*θ*_Manus_ = *θ*_Lau_ = *θ*_Ancestral_								
21—unconstrained migration rates	−1,099	2	2,204***	4.1820	[4.1820]	[4.1820]	0.3109	0.5203
22—equal migration rates	−1,108	3	2,223***	4.1820	[4.1820]	[4.1820]	0.3985	[0.3985]
23—no migration from Lau to Manus	−1,556	3	3,118***	4.5900	[4.5900]	[4.5900]	[0.00000]	0.7276
24—no migration from Manus to Lau	−1,882	3	3,771***	4.5764	[4.5764]	[4.5764]	0.4581	[0.00000]
25—no migration	−3,191	4	6,389***	4.5395	[4.5395]	[4.5395]	[0.00000]	[0.00000]

Note

Migration parameters, *m*_Lau > Manus _(Lau to Manus) and *m*_Manus > Lau_ (Manus to Lau), are given in forward time (i.e., reversed from the backward coalescent time given by IMa2) to facilitate comprehension. *p*‐values obtained by comparing 2LLR to a *χ* ^2^ distribution at the *df* degree of freedom: *^NS^*, *p* > 0.20; *, *p* < 0.05; ***, *p* < 0.001

Upper‐bound priors of the uniform distribution (*t* = 2, *θ* = 50 for all populations, and *m* = 5) and heating regimes among chains were chosen after multiple pilot runs. An infinite site model of substitution was chosen in accordance with the IMgc filtering that produced nonrecombining blocks of sequence. To achieve good swapping among the 80 Markov implemented chains, a geometric increment model of heating was used (−ha 0.96 −hb 0.9). After 400,000 steps of burn‐in, 10,000,000 additional steps were performed; recording parameter estimates every 10 steps. Convergence was assessed using multiple runs, and marginal likelihood parameter estimations for the best performing run are presented. From this best M‐mode run, a total of 100,000 genealogies were recorded and loaded in L‐mode run for comparison with each nested demographic model. L‐mode reports log‐likelihood ratio (LLR) tests to identify poorer fitting models.

### 2.8 Migrate‐N analysis

Migrate‐n was run according to the supplied guide (Beerli, 2009; Beerli & Felsenstein, 1999; Beerli & Palczewski, 2010). Briefly, we conducted a maximum likelihood inference starting with the default parameter file supplied with Migrate‐n v3.6. A second run was then conducted using the parameters estimated in run 1 as the starting values. A third run was conducted using the parameters from run 2 as the starting values and using longer chains (short chain = 5,000 steps and long chain = 50,000 steps) and a larger sampling increment (increment = 1,000). The parameter estimates from run 2 and run 3 were consistent, and the estimates from run 3 are reported here.

## 3 RESULTS

### 3.1 Allelic reconstruction and data filtering produced 42 amplified loci

Sequences for a total of 42 PCR‐amplified nuclear intronic and exonic loci averaging 237 bp were generated for population analysis of 93 individuals (Table 2). Specifically, 16 loci were composed exclusively of introns (after trimming), 9 loci were exclusively exons, and the remaining 17 loci contained both intronic and exonic regions. Exclusively exonic amplicons were the result of unsuccessful intron prediction but were still informative enough to include in the analysis. PCR amplification success (individuals per locus) ranged from 26% to 100%, with a median of 91%. The range of median number of polymorphic sites per locus was 23, ranging from 2 to 60 sites. No loci were discarded from our analysis due to missing data since IMa2 analysis has been shown to be more robust with more loci despite missing data (Hey, Chung, & Sethuraman, 2015).

### 3.2 Species analysis reveals the presence of only *L*. aff. *schrolli*

We used COI sequences to determine that the species in this study belong to the *L*. aff. *schrolli* species designation. We were able to obtain COI sequences for 81 individuals using Sanger sequencing and 71 individuals using the 454 sequenced PCR products. Two individuals did not have sequences long enough for further analysis but both of these had reciprocal best Blastn hits (Altschul et al., 1990) to *L*. aff. *schrolli* isolated from the Lau and Fiji basins. Three individuals (TM.98, SW8.3514, and SW1.97) did not amplify using either technique. Nuclear data for these three individuals were not divergent from the other *L*. aff. *schrolli* individuals. Therefore, it is reasonable to assume they belong to the same species complex.

Assembly of the COI loci sequenced using 454 suggested that there is significant heteroplasmy within this species complex. Twenty‐one individuals had two distinct mitochondrial COI haplotypes. Mitochondrial heteroplasmy has previously been detected between *L. elevatus north* and *L. elevates south* (Plouviez et al., 2009). Analysis with Mothur identified 3 OTUs with at least 3% divergence in the analysis of both the Sanger sequenced and 454 sequenced COI loci—all individuals were assigned to the same OTU in both analyses. One OTU contained all the *L*. aff. *schrolli* sequenced from the Mariana Trough (EU306431‐436), the second OTU contained all the *L. schrolli* sequenced from the Manus Basin (EU306437‐442), and the final OTU contained all the individuals sampled in this study and the *L*. aff. *schrolli* collected from the Fiji and Lau Basins (EU306451‐456). Median‐joining haplotype networks of these individuals are shown in Figures S1 and S2.

### 3.3 Population structure analysis reveals two divergent populations

**Figure 3:**
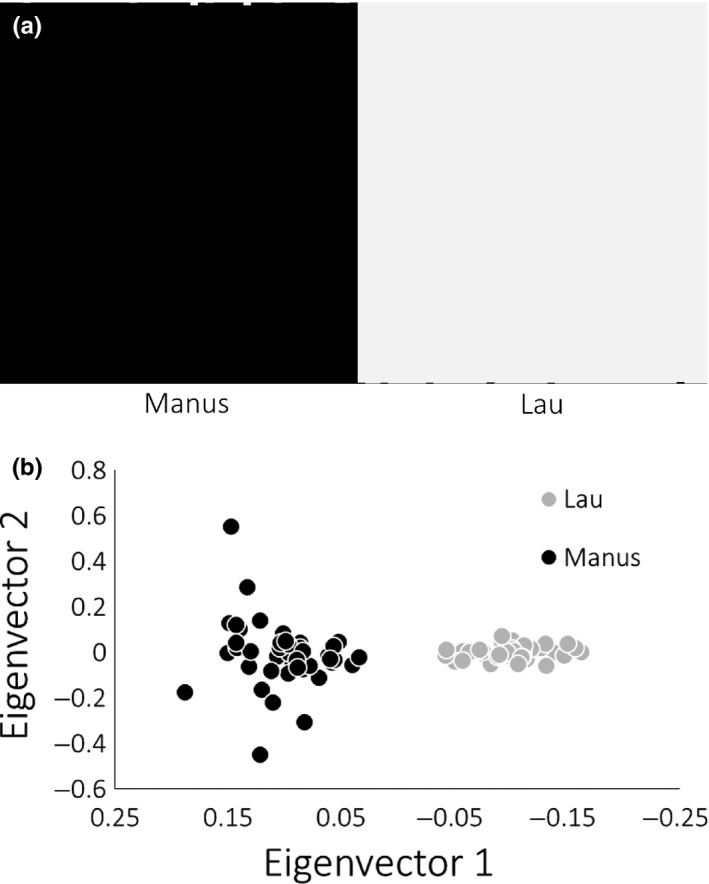
Structure assignment plot clustering Manus and Lau basins into two distinct genetic groups (a), PCA of between‐basin variance in *Lepetodrilus* aff. *schrolli* (b). Manus and Lau individuals are represented in black and gray, respectively

Using the no‐admixture model, two distinct genetic groups (*K* = 2, Figure 3a) were identified by Structure 2.3.4. (Hubisz et al., 2009; Pritchard et al., 2000) and assessed by the Δ*K* statistic (mean ln *p*(*D*) = −10387, similarity score = 0.998). These two groups corresponded to individuals sampled from the Manus and Lau basins. Each individual was probabilistically assigned to a basin‐specific cluster with a posterior probability greater than 0.98. The high posterior probability value for each individual suggested that the no‐admixture model (which is more appropriate for discrete populations) fits our data and that none of these individuals were outliers. Outliers in the nonadmixed model may suggest migrants and/or hybrids (Pritchard et al., 2000). Similar results were produced when using the admixture model (results not shown), indicating that each individual inherited almost the entirety of its genome from the basin where it was sampled, and that recent migrants and/or hybrids are absent from our samples.

PCA analysis clustered individuals by basin along eigenvector 1 with a correlation of −0.947 (Figure 3b). Overall, population differentiation between basins along the first eigenvector was highly significant (*p*‐value = 5.4 × 10^−50^: summed across AMOVA comparisons).

### 3.4 Isolation and migration analysis reveals gene flow between divergent populations

**Figure 4:**
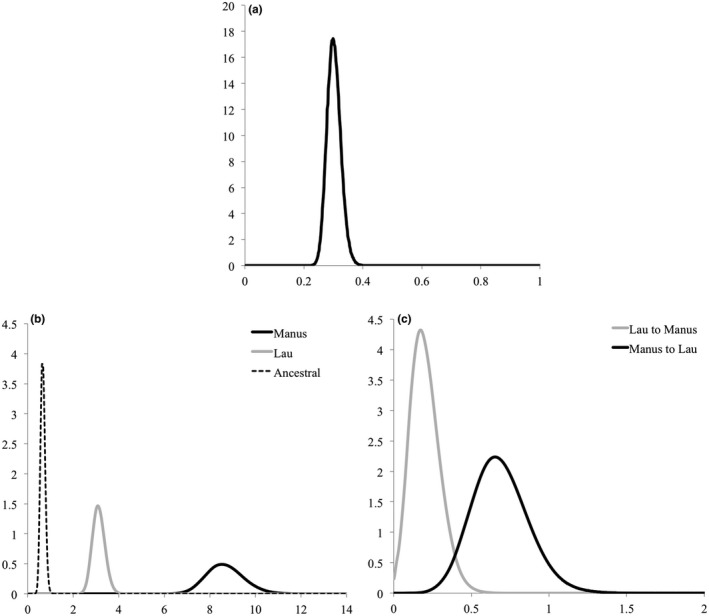
IMa2p marginal distributions for the population splitting time *t* (a), the effective population sizes *θ* (b), and the migration rate parameters in forward time *m* (c)

The unconstrained model, M‐mode in IMa2p, estimated a population splitting time parameter *t* of 0.29 (HiPt = 0.299, HPD95Lo‐Hi: 0.253–0.351). Population parameters *θ* differed among populations (Figure 4, Table 3), with *θ_ancestral_* < *θ_Lau_* < *θ_Manus_*. Marginal posterior distributions of *θ* for each population were nonoverlapping (Figure 4). Forward in time, migration rates from Manus to Lau and from Lau to Manus were nonzero in M‐mode (Figure 4, Table 3). The migration rate from Manus to Lau was more than 3 times higher than in the opposite direction (*m*_Manus→Lau_ HiPt = 0.6525, HPD95Lo‐Hi: 0.3375–1.048; *m*_Lau→Manus_ *_s_* HiPt = 0.1725, HPD95Lo‐Hi: 0.0325–0.3925). Marginal posterior distributions overlapped between the two migration parameters (Figure 4), but the probability of *m*_Manus→Lau_ > *m*_Lau→Manus_ was 0.983.

When compared with all possible nested‐models in L‐mode (Table 3), only one model was not rejected: the model of no migration from Lau to Manus (model 3: *p* > 0.2). This model had unconstrained *θ* population parameters with a probably of 0.983 that *θ_ancestral_* < *θ_Lau_*, *θ_ancestral_* < *θ_Manus_*, and *θ_Lau_* < *θ_Manus_*. All models testing for “no migration from Manus to Lau” were rejected (*m*_Manus→Lau_ > 0). The equal migration rate model (model 2, *p* < 0.05) was also rejected. However, model 3 of “no migration from Lau to Manus” could not be rejected, indicating a low or null *m*_Lau→Manus_. According to both the MCMC and L‐modes analyses, isolation between the two basins was followed by low rates of migration, with a higher migration rate from Manus to Lau.

### 3.5 Migrate‐n analysis supports gene flow between differentiated populations

Migrate‐n is frequently used to estimate migration among populations and effective population sizes. The software is based on a model of constant migration rates and effective population sizes over time, with no history of population splitting or other demographic changes. Simulations result in inaccurate estimates of migration rates under violations of this assumption, especially when the populations have recently split (see the migrate‐n website: http://popgen.sc.fsu.edu/Migrate/Migrate-n.html). The migrate‐n results from the 42 loci predict a migration rate from Manus to Lau that is three and a half times higher than the migration rate from Lau to Manus. The analysis also reveals an effective population size in Manus that is twice as large as that in Lau (Figure S3). However, since a non‐null splitting time was found using IMa2p, we are likely violating the assumptions of Migrate‐n. Nevertheless, the results from Migrate‐n are consistent with those of IMa2p.

## 4 DISCUSSION

In the Lau and Manus basins of the Southwest Pacific, the limpet *L*. aff. *schrolli* does not show significant within‐basin population structure. Between basins, there is significant population differentiation in *L*. aff. *schrolli* —including a lack of first‐ or second‐generation migrants. Coalescent analysis of 42 single‐copy amplicons, however, reveals that this strong population structure does not preclude the presence of gene flow between distant populations after isolation. This finding suggests that populations of vent organisms that have previously been reported to be isolated between basins may have significant gene flow between basins that can be revealed using more loci in a coalescent framework.

We did not detect any population differentiation between vents within basins for *L*. aff. *schrolli* (Figure 3). This is not surprising given that in previous studies investigators have not detected within‐basin population structure in Maus nor Lau (Table 1) except in the case of *M. lauensis* (Thaler et al., 2014). Analysis with Structure 2.3.4 did not detect first‐ or second‐generation migrants which provides further evidence for population differentiation between Manus and Lau basins. This is consistent with the differentiation detected between populations of *M. lauensis* in Manus and Lau basins (Thaler et al., 2014). Additionally, between‐basin differentiation is consistent with studies that show differentiation between populations found in Manus and North Fiji for *I. nautilei, Chorocaris* sp. 2, and *Bathymodiolus* (Kojima et al., 2000; Kyuno et al., 2009; Thaler et al., 2014, 2011). So far, only two subspecies of *Alviniconcha* have lacked between basin population differentiation between Manus and North Fiji (Kojima et al., 2001; Suzuki et al., 2006). In addition to detecting population structure, we were simultaneously able to detect a low rate of gene flow between Manus and Lau in *L*. aff. *schrolli*.

Population structure, detected by genetic differentiation between the two basins, does not exclude the possibility of past or present gene flow. The amplicon sequence data fit a coalescence model of isolation with migration between the Manus and Lau basin populations. Migration between Manus and Lau was inferred to be either entirely eastward (Manus → Lau) or predominately eastward with some westward migration (Lau → Manus). Population isolation between basins with some migration is consistent with the nonequilibrium model (tested in IMa2p).

The IMa2p model of isolation with migration does not differentiate between ongoing migration (after isolation) and a colonization event (inferred migration corresponding to traces of this colonization prior to the isolation). IMa2p could confound the effects of colonization from Manus to Lau with migration from Manus to Lau (Grosberg & Cunningham, 2001; Slatkin, 1993). The significantly smaller *θ* in the Lau basin may be consistent with such an eastward colonization event, but addressing this question would require sampling additional locations or explicit tests of both models. In both colonization and migration scenarios, past gene flow has been predominantly or exclusively eastward.

To determine whether our observations are consistent with oceanographic data, we compared our findings with simulations of connectivity in the region. First, Suzuki et al. (2018) predicted dispersal between vents within both the Manus and Lau basins, but no dispersal directly between any two vents between the Manus and Lau basins. Additionally, the mean recovery time of vents near Solwara 1 and Solwara 8 is 3–5 times longer than those in Lau—this implies limited connectivity flowing into the Manus Basin (Suzuki et al., 2018). Second, we compared our results to Mitarai et al. (2016) who estimated the rate and direction of potential larval dispersal among western Pacific back‐arc basins, including our study sites and the basins that lie between them (Figure 2). Assuming a relatively long pelagic larval duration (PLD) of 170 days at a depth of 1,000 m, Mitarai et al. (2016) concluded that all basins in the region could be connected by larvae via stepping‐stones. This dispersal, however, is predicted to be exclusively westward between the Solomon and Woodlark basins (Figure 2). For these basins to serve as stepping‐stones for migration from Manus to Lau, larvae would need to be transported eastward. Furthermore, some of these stepping‐stones may only be connected by larvae once every hundred thousand years. Similar results were found with the more rapid currents at 500 m with a shorter PLD of 90 days. When the PLD was shortened to 83 days at 1,000 m or 43 days at 500 m, no dispersal was predicted between Woodlark/Solomon and Solomon/North Fiji, breaking the stepping‐stone chain between Manus and Lau.

Gene flow between Manus and Lau could result from a long PLD for *L*. aff. *schrolli*, ocean current anomalies, or even unknown vent sites that serve as stepping‐stones. For mollusks that inhabit deep‐sea chemosynthetic environments, estimates of PLD are scarce. The PLD for the planktotrophic larvae of *Bathymodiolus childressi* was estimated to be up to 13 months in *B. childressi* mussels (Arellano & Young, 2009) and over 8 months in *Bathynerita naticoidea* snails (Van Gaest, 2006). It has been suggested that colder deep‐sea temperatures and limited food availability lowers the metabolism of lecithotrophic larvae facilitating long PLDs and dispersal distances (Young, Sewell, Tyler, & Metaxas, 1997). *Lepetodrilus* spp. larvae are generally considered lecithotrophic (Berg, 1985; Craddock, Lutz, & Vrijenhoek, 1997; Tyler et al., 2008), even though their small oocytes are comparable with those of species with planktotrophic larvae (Tyler et al., 2008). Furthermore, *Lepetodrilus* limpets can live in habitats peripheral to vents where they may rely on grazing (Bates, 2007), albeit in lower densities (Bates, Tunnicliffe, & Lee, 2005). Due to the low density of nonvent populations, it is unlikely they contribute significantly to gene flow between densely populated vent sites.

Our analyses suggest that genetic differentiation between Manus and Lau detected in other species using COI and/or microsatellite markers (Table 1) does not exclude the possibility of gene flow between basins in those species. Using 42 sequenced gene regions in a coalescent framework allowed us to infer directional gene flow in the presence of genetic differentiation for *L*. aff. *schrolli*. Sampling additional Western Pacific basins, such as Woodlark, Solomon, New Hebrides, and North Fiji, would provide a better understanding of connectivity in the region. Furthermore, a timescale for divergence between populations in the Manus and Lau basins could be obtained by expanding amplicon sequencing to a pair of limpet species where the divergence time can be estimated based on geologic events. For example, the limpet species *Lepetodrilus fucensis* and *Lepetodrilus gordensis* were putatively separated by the formation of the Blanco Transformation Fault between 5 and 18 MY ago (Johnson et al., 2006). Amplicon sequences from the same loci in *L. fucensis* and *L. gordensis* would allow us to convert the IMa estimates of divergence in generations to years.

Based on the oceanographic currents model by Mitarai et al. (2016), we predict *L*. aff. *schrolli* in Manus and Woodlark Basins are genetically connected while also differentiated from a New Hebrides‐North Fiji‐Lau Basin group. Samples from Solomon could help determine the mechanism behind differentiation between Manus and Lau in the presence of gene flow. Nevertheless, this analysis suggests that gene flow occurs within the Western Pacific hydrothermal vent fields: We detect historical gene flow between Manus and Lau and expand the species range of *L. *aff. *schrolli* to include the Manus Basin. This conclusion, however, cannot be extended to other species without further analysis. Estimates of gene flow and population connectivity could be used to predict possible species loss due to mining in the region. With 42 loci, the present study is also one of the very few marine population studies using >10 nuclear loci in a coalescent theory framework (Hurt, Silliman, Anker, & Knowlton, 2013; Jang et al., 2016; Smith et al., 2015; Weber, Merigot, Valiere, & Chenuil, 2015). Given the breadth and depth of this study, it should serve a case study for future work on phylogeography in marine habitats.

## CONFLICT OF INTEREST

None declared.

## AUTHOR CONTRIBUTIONS

S.P., A.L.L., J.E.N., C.L.V.D., and C.W.C. designed research; S.P. and B.B. designed and amplified primers for all genes/individuals; A.L.L designed pipeline to physically link alleles; S.P., A.L.L., F.A.B.v.M., J.E.N., and C.W.C. analyzed the data; all the authors wrote the manuscript.

## Data Availability

Amplicon sequences are uploaded to GenBank. Accession numbers can be found in Table S1. Scripts, where appropriate, will be uploaded to the github of A.L.L. https://github.com/alabella19/Plouviez_et_al_2019.
