# Supplementary material for: Amplicon sequencing of 42 nuclear loci supports directional gene flow between South Pacific populations of a hydrothermal vent limpet
Source: Ecol Evol. 2019 May 6;9(11):6568–80. doi: 10.1002/ece3.5235 (PMC6609911; doi:10.1002/ece3.5235)
Supplement: Supplementary file 1 [file ECE3-9-6568-s001.docx]

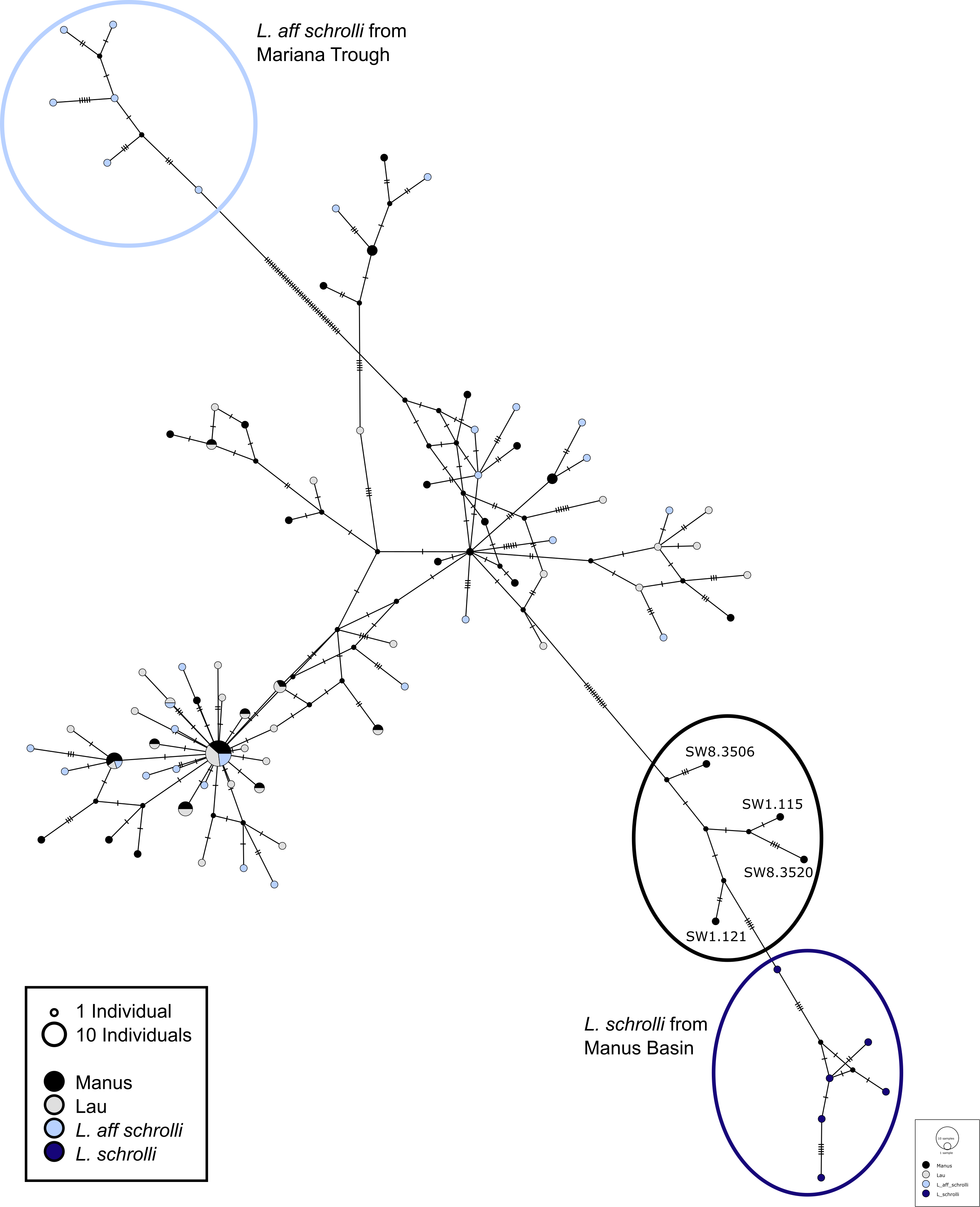


Supplementary Figure 1: Median-Joining tree of Sanger sequenced COI loci form our study compared to those from Johnson et al. (2008). All of our samples cluster with *L.* aff *schrolli* collected by Johnson et al. (2008) from the Fiji and Lau Basins (light blue). The four individuals in the black circle are the most distant from *L.* aff *schrolli* but OTU analysis assigns them to the OTU containing *L.* aff. *schrolli*


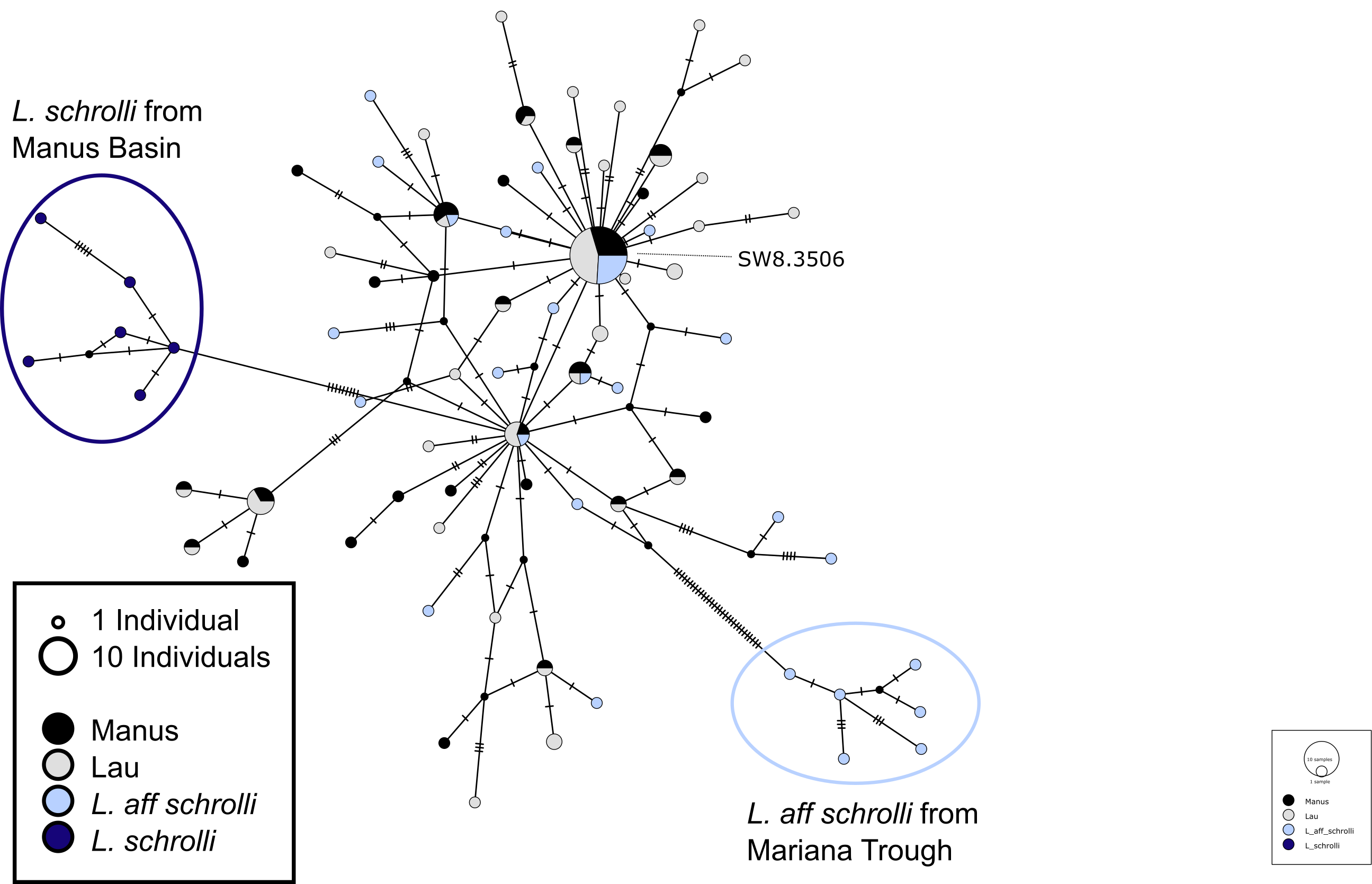


Supplementary Figure 2: Median-Joining tree of 454 sequenced COI loci from our study compared to those from Johnson et al. (2008). All samples collected in this study cluster with *L.* aff *schrolli* collected from the Lau and Fiji Basins (light blue).

A)


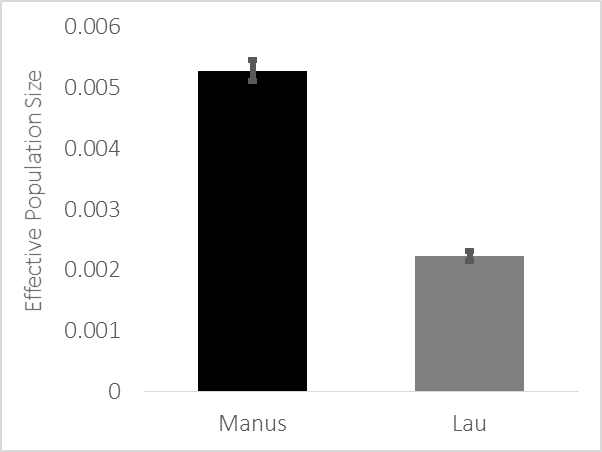


B)


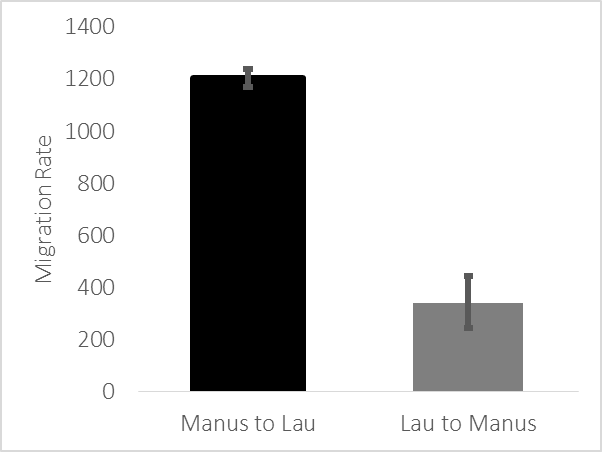


Supplementary Figure 3: Migrate-n results from the 42 loci. A) Effective population sizes estimated by migrate-n show an effective population size in Manus that is twice as large as that in Lau. B) Migration rate from Manus to Lau is estimated in Migrate-n to be over three times larger than that in the opposite direction.

Johnson SB, Waren A, Vrijenhoek RC (2008) DNA Barcoding of Lepetodrilus Limpets Reveals Cryptic Species. *Journal of Shellfish Research* **27**, 43-51.
