# Supplementary material for: Amplicon sequencing of 42 nuclear loci supports directional gene flow between South Pacific populations of a hydrothermal vent limpet
Source: Ecol Evol. 2019 May 6;9(11):6568–80. doi: 10.1002/ece3.5235 (PMC6609911; doi:10.1002/ece3.5235)
Supplement: Supplementary file 2 [file ECE3-9-6568-s002.docx]

Supplementary table 1: Locus information for *Lepetodrilus* aff *schrolli* amplicons

| Locus | Primers  Forward/Reverse | Genbank Accessions | *Drosophila melanogaster* ortholog | Type of loci | Gene Copies | No. Poly-morphic Sites | Length (bp) | % of total individuals with at least 1 allele |
| --- | --- | --- | --- | --- | --- | --- | --- | --- |
| Le60RibProtL8Ex | AAATCGGCAACGTTCTTCC/  AACCATTGCCCTGTTAGTCG | MK142923 - MK143104 | ribosomal protein L8, isoform A | Exon | 182 | 5 | 170 | 98% |
| Le60SRpL13Ex | TGGCTCCCAAAAGAAATAACA/  GTGAAAATCCACGGCCAGAC | MK222101 - MK222282 | ribosomal protein L13, isoform A | Exon | 182 | 15 | 190 | 98% |
| LeAconEx | TTGTCTGACCTAGGAGGTGTG/  CATCACTTCCCACAAGTTCG | MK221917 - MK222100 | aconitase, isoform B | Exon | 183 | 18 | 186 | 99% |
| LeAconInt2FW | GAGCCGGTCAGCCAGTATC/  GGACCACACCTCCTAGGTCA | MK221733 - MK221916 | aconitase, isoform B | Exon/Intron | 165 | 45 | 163 | 98% |
| LeAconIntFW | GTGAATGGCCCCTTTACCC/  GTTTGGCGATACTGGCTGAC | MK221565 - MK221732 | aconitase, isoform B | Exon/Intron | 139 | 40 | 185 | 80% |
| LeActBIntFW | CTTGCCGAATCCAAGGAATA/  TGGCTCGTGGTTATCGATCT | MK221385 - MK221564 | beta spectrin, isoform B | Intron | 162 | 46 | 331 | 94% |
| LeATPsyntint | TGCCAGAGACCAGTGGTAAA/  AATAGGCAACCAAGCCAGTG | MK221245 - MK221384 | ATP synthase, subunit B | Exon/Intron | 131 | 29 | 799 | 75% |
| LeEF1Int3FW | CTCTTCCCGGTGACAATGTT/  ATTTGACCAGGGTGGTTCAG | MK220908 - MK221083 | elongation factor 1 | Exon/Intron | 171 | 53 | 400 | 94% |
| LeEF1Int | CCCAACAACATCACCACTGA/  GGCAGAGCCTCAGTCAGAGT | MK221084 - MK221244 | elongation factor 1 | Exon/Intron | 147 | 60 | 421 | 86% |
| LeEF2Int2 | GAAGACCGGTACCATCTCCA/  GCTGGGGTTCTTGCACTCTA | MK220728 - MK220907 | elongation factor 2 | Intron/Exon | 175 | 19 | 182 | 97% |
| LeEnolInt2 | CAACTGCTTGCTGCTCAAAG/  TGAAGGTGTCCTCGGTCTCT | MK220668 - MK220727 | Enolase, isoform A | Exon/Intron/Exon | 57 | 20 | 237 | 31% |
| LeFruBiEx | GCTGACGCAGATTGGTGTAG/  GACTCCCTTGTCCACCTTGA | MK220488 - MK220667 | Fructose-1,6-Bisphosphate Aldolase | Exon | 164 | 14 | 158 | 96% |
| LeFruBiInt3FW | GCCCATTGTTGAACCAGAAG/  TACGTTGTGATCTGCCAAGG | MK220402 - MK220487 | Fructose-1,6-Bisphosphate Aldolase | Intron | 75 | 36 | 277 | 42% |
| LeFruBiIntFW | TCAAGGTGGACAAGGGAGTC/  TCCTTTTTGTACTGGGCACAC | MK220226 - MK220401 | Fructose-1,6-Bisphosphate Aldolase | Intron | 160 | 36 | 187 | 91% |
| LeGlyPInt3 | CCCAGGCTATGGAAACAACA/  GCAACATTGCGATCACAGAC | MK220048 - MK220225 | glycogen phosphorylase, isoform A | Intron | 178 | 15 | 165 | 96% |
| LeGlyPIntFW | GGCTACGGGATCAGATACGA/  TTGATGGGCAGCATGTACTC | MK219910 - MK220047 | glycogen phosphorylase, isoform A | Intron/Exon | 122 | 31 | 318 | 71% |
| Locus |  | Genbank Accessions | *Drosophila melanogaster* ortholog | **Type of loci** | Gene Copies | No. Poly-morphic Sites | Length (bp) | % of total individuals with at least 1 allele |
| LeGpoInt2 | GAGAGCATGGGTTTACTGGA/  GAGGGTAGTCGGTCACCTCA | MK219738 - MK219909 | glycerophosphate oxidase-1, isoform C | Intron | 166 | 17 | 287 | 91% |
| LeGpoInt3 | GAGGTGACCGACTACCCTCA/  CAGCTACCAGAGGTCGAAGG | MK219574 - MK219737 | glycerophosphate oxidase-1, isoform C | Exon/Intron | 162 | 19 | 305 | 87% |
| LeGTPIntFW | CCTTGTCAATCTCTCTCAAAAAG/  GGTCATTAGCGTCGATCCAT | MK219388 - MK219573 | CG1354, isoform B (YchF-GTPase) | Intron | 185 | 24 | 474 | 100% |
| LeGuaNuExFW | CTCGTCACTTCCGAGACCAT/  GTCCCAGCCACAGGACAC | MK219212 - MK219387 | G protein beta-subunit 13F, isoform A | Exon/Intron/Exon | 158 | 37 | 261 | 92% |
| LeGuaNuInt | TTTGCTCTGTCTTCGTCGTG/  TGGTCTCGGAAGTGACGAGT | MK219066 - MK219211 | G protein beta-subunit 13F, isoform A | Intron | 130 | 19 | 189 | 77% |
| LeLethalInt | GACGGCACCACAAAGAAAAT/  GCCAGAATCTGTACGGAGGA | MK218882 - MK219065 | ATP synthase, delta subunit, isoform A | Intron | 172 | 23 | 180 | 99% |
| LemtsInt | AACAGTTTCGCTTCTAGTTGGTTT/  GCGCAAACACTCGTCATAAA | MK218708 - MK218881 | microtubule star, isoform A | Intron/Exon | 173 | 21 | 245 | 94% |
| LeMyoHeaCInt | CACCATCGACAAACTCAACG/  CTCCAGCTCATTCTGCACCT | MK218538 - MK218707 | myosin heavy chain, isoform P | Exon/Intron/Exon | 157 | 23 | 148 | 91% |
| LeNudCInt | CAACGAACAGCAGAAACGTC/  ATGCTTGTCTCAGCATGTCG | MK218422 - MK218537 | nudC isoform A | Exon | 116 | 17 | 143 | 62% |
| LePepIsoIntFW | CAAGCAAAGAATGCACTCCA/  GCTGACATTTTAACGCTGCTT | MK218270 - MK218421 | P-element somatic inhibitor, isoform A | Intron | 150 | 14 | 89 | 81% |
| LePGMInt3 | ACCCACAAGTCCGTGGAAG/  GCTGGCACAGATTCACAGTT | MK218088 - MK218269 | phosphoglucose mutase | Exon/Intron | 153 | 22 | 78 | 97% |
| LePGMIntFW | GGTCAAAGGTTATGCCAGGA/  CAGCATCCATCAGGTTACCA | MK217950 - MK218087 | phosphoglucose mutase | Intron/Exon | 106 | 11 | 79 | 63% |
| LePolyApolInt | GAATGACATTGCGTTCAATCC/  CCCAGCACGTTGGAGTAGAT | MK223529 - MK223710 | Poly(A) polymerase | Intron | 180 | 38 | 155 | 98% |
| LeProt1IntFW | TGGTGTTATCATGGGAGCTG/  CACCTTCGTCAGCTTGTCTG | MK223427 - MK223528 | proteasome beta1 subunit | Intron | 83 | 21 | 93 | 51% |
| LeProtDiSulfIntFW | GGATTGGGATGCTAAACCAG/  CCAGATAGGAGCCAGCTGTT | MK223279 - MK223426 | protein disulfide isomerase, isoform A | Exon/Intron | 119 | 24 | 172 | 72% |
| LePSomInhIsoAInt | CATTGGGAAAGGTGGAGAAA/  TGGCGCATTACTGTCTTGAA | MK223117 - MK223278 | P-element somatic inhibitor, isoform C | Exon/Intron/Exon | 149 | 35 | 441 | 86% |
| LeRab1Ex | TGCAGACCAACTAGGCATCC/  GGGGTGCTCGAGTTAATTTTT | MK222991 - MK223116 | Rab1 | Exon | 124 | 2 | 57 | 67% |
|  |  |  |  |  |  |  |  |  |
| Locus |  | Genbank Accessions | *Drosophila melanogaster* ortholog | **Type of loci** | Gene Copies | No. Poly-morphic Sites | Length (bp) | % of total individuals with at least 1 allele |
|  |  |  |  |  |  |  |  |  |
| LeRabProt7Ex | CAAAGCGGGCTCACAGTT/  TTAACAGCTGCATCCATCC | MK224225 - MK224406 | Rab7, isoform A | Exon | 182 | 7 | 156 | 98% |
| LeRiboD3Ex | GCGTGCCGATTAAAGTTCTG/  GCACGAAGAATGGCAGATTT | MK222811 - MK222990 | small ribonucleoprotein particle protein SmD3 | Exon | 180 | 6 | 259 | 97% |
| LeRiboD3Int | GGAGAGGGAAATCTGCCATT/  CGTCGCTTTTGGAAAACATT | MK222627 - MK222810 | small ribonucleoprotein particle protein SmD3 | Intron | 158 | 25 | 229 | 94% |
| LeRieskEx | CTTCAACGTATGCAGCCAAG/  ACCCAACAGAATCAGCCACT | MK222467 - MK222626 | rieske iron-sulfur protein, isoform B | Exon | 157 | 21 | 236 | 86% |
| LeRNArecIntFW | GAGACAACAAGCATTGGCATT/  AGGCTTGCTTGATGGTGTCT | MK222283 - MK222466 | half pint, isoform A (wih 3 RNA recognition motifs) | Intron | 184 | 25 | 267 | 99% |
| LeSplFIntFW | GGCATCCGTTAACTCGCTAC/  TCCGAGAACAGTGCATGGTA | MK223711 - MK223768 | caper, isoform B | Intron | 45 | 25 | 563 | 26% |
| LeThioEx | AGTGCAAAATACCCGAAAGC/  CTTTGACAGCCACCTCATCA | MK223769 - MK223950 | Thioredoxin-like | Intron/Exon | 163 | 26 | 206 | 97% |
| LeUbiqIntFW | CATTCTTTGCCTTCTCAGAACC/  AAGCGGTCCCACAGAGTG | MK223951 - MK224044 | ubiquitin activating enzyme 1, isoform A | Intron | 83 | 29 | 111 | 47% |
| LeVarSupInt | GTCCCGGGAGGTCTCATT/  CACAGAGTGGGGTCAAACTTC | MK224045 - MK224224 | suppressor of variegation 3-9 | Intron | 159 | 35 | 197 | 90% |
