# Supplementary figures and images for: Amplicon sequencing of 42 nuclear loci supports directional gene flow between South Pacific populations of a hydrothermal vent limpet
Source: Ecol Evol. 2019 May 6;9(11):6568–80. doi: 10.1002/ece3.5235 (PMC6609911; doi:10.1002/ece3.5235)

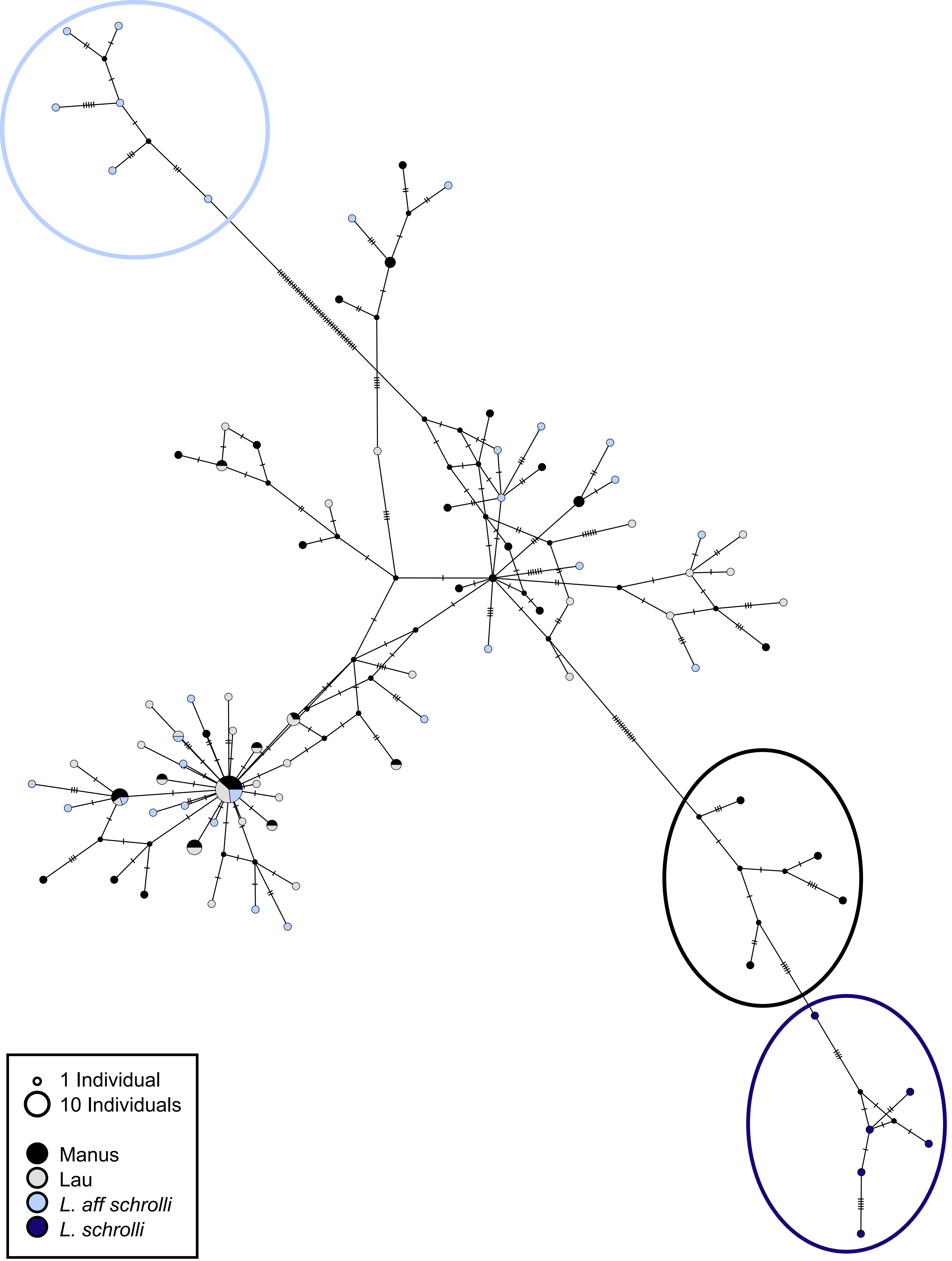

Supplement: Supplementary file 3 [file ECE3-9-6568-s003.pdf]

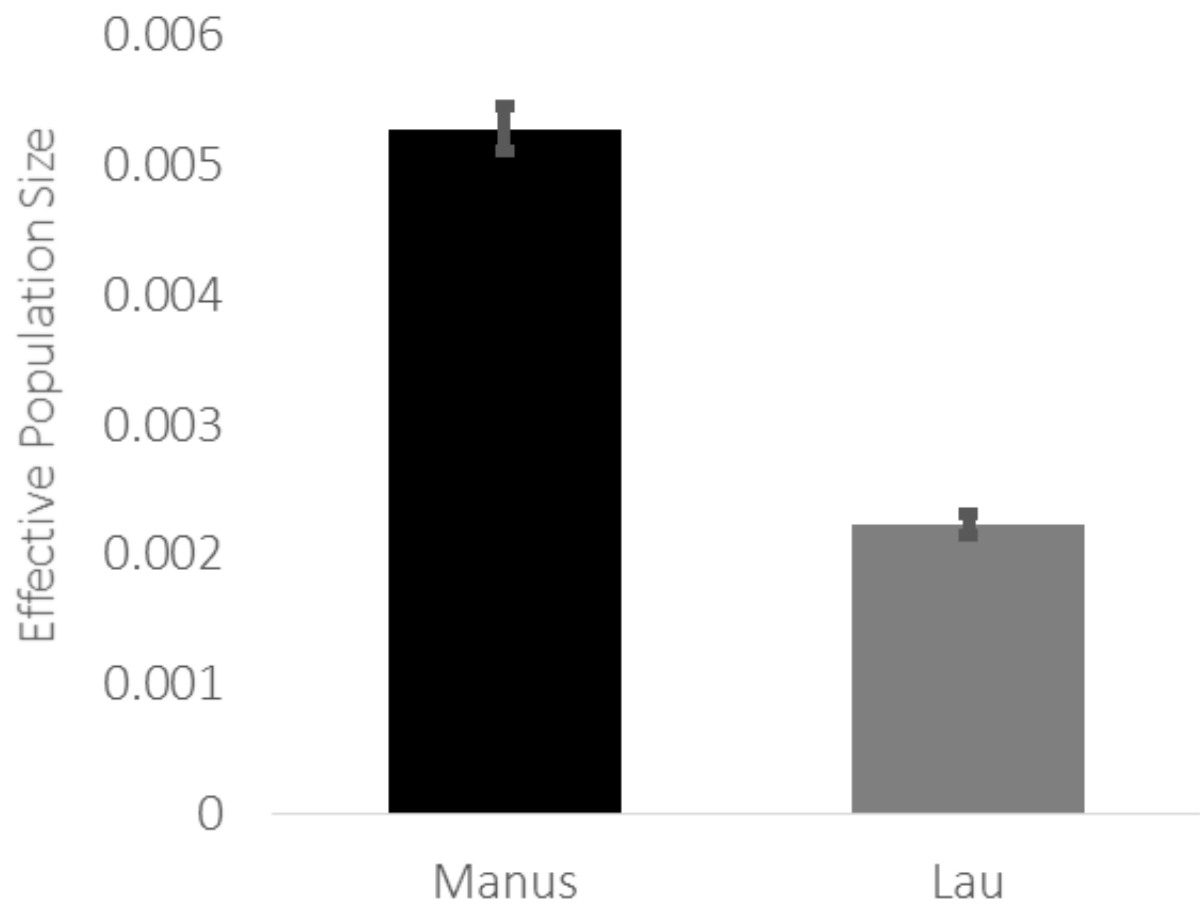

Supplement: Supplementary file 5 [file ECE3-9-6568-s005.pdf]
