# Supplementary material for: Amplicon sequencing of 42 nuclear loci supports directional gene flow between South Pacific populations of a hydrothermal vent limpet
Source: Ecol Evol. 2019 May 6;9(11):6568–80. doi: 10.1002/ece3.5235 (PMC6609911; doi:10.1002/ece3.5235)
Supplement: Supplementary file 4 [file ECE3-9-6568-s004.pdf]

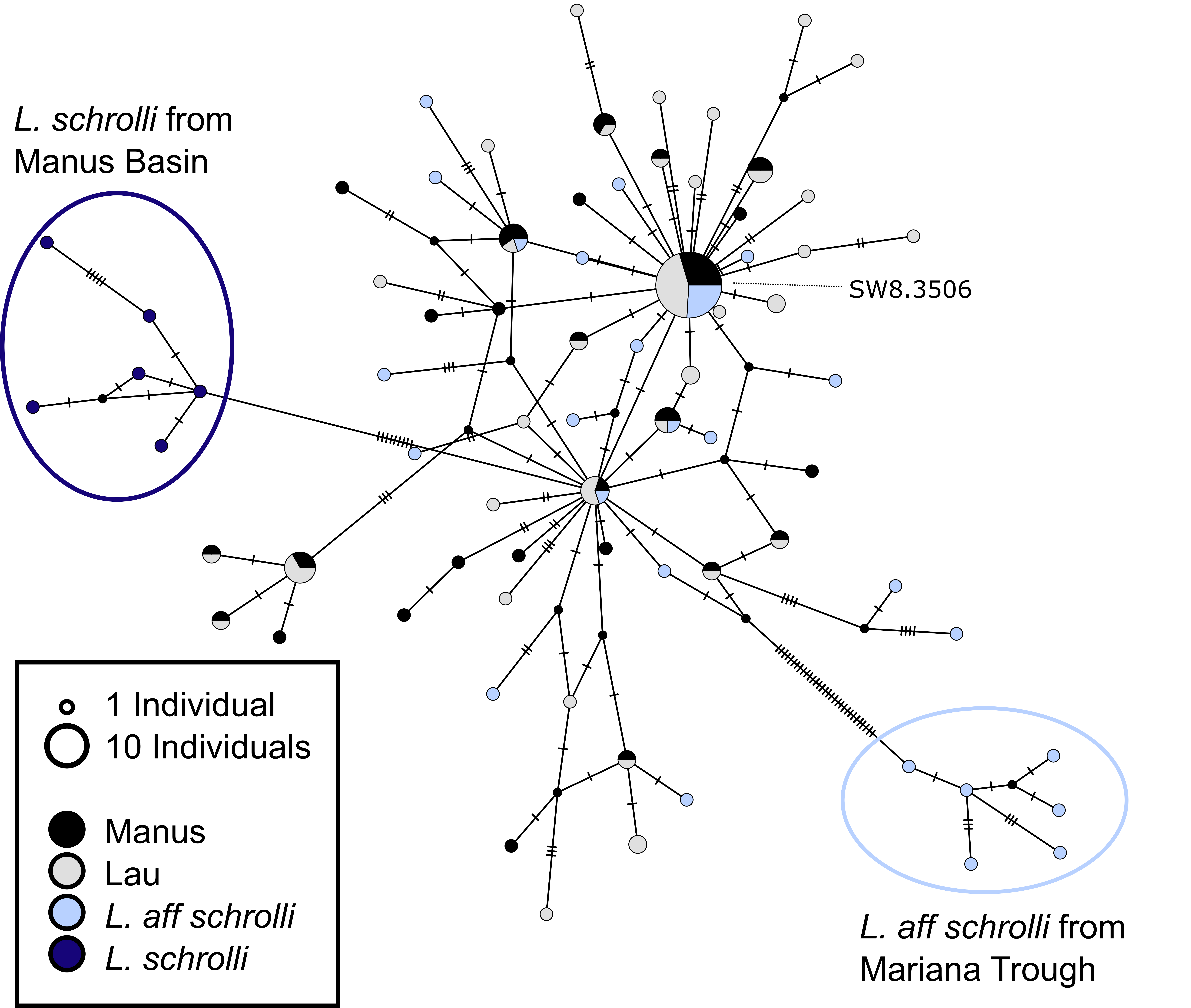
L. schrolli from
Manus Basin
SW8.3506
1 Individual
10 Individuals
Manus
Lau
L. aff schrolli
L. schrolli
L. aff schrolli from
Mariana Trough
