# Supplementary material for: Amplicon sequencing of 42 nuclear loci supports directional gene flow between South Pacific populations of a hydrothermal vent limpet
Source: Ecol Evol. 2019 May 6;9(11):6568–80. doi: 10.1002/ece3.5235 (PMC6609911; doi:10.1002/ece3.5235)
Supplement: Supplementary file 6 [file ECE3-9-6568-s006.pdf]

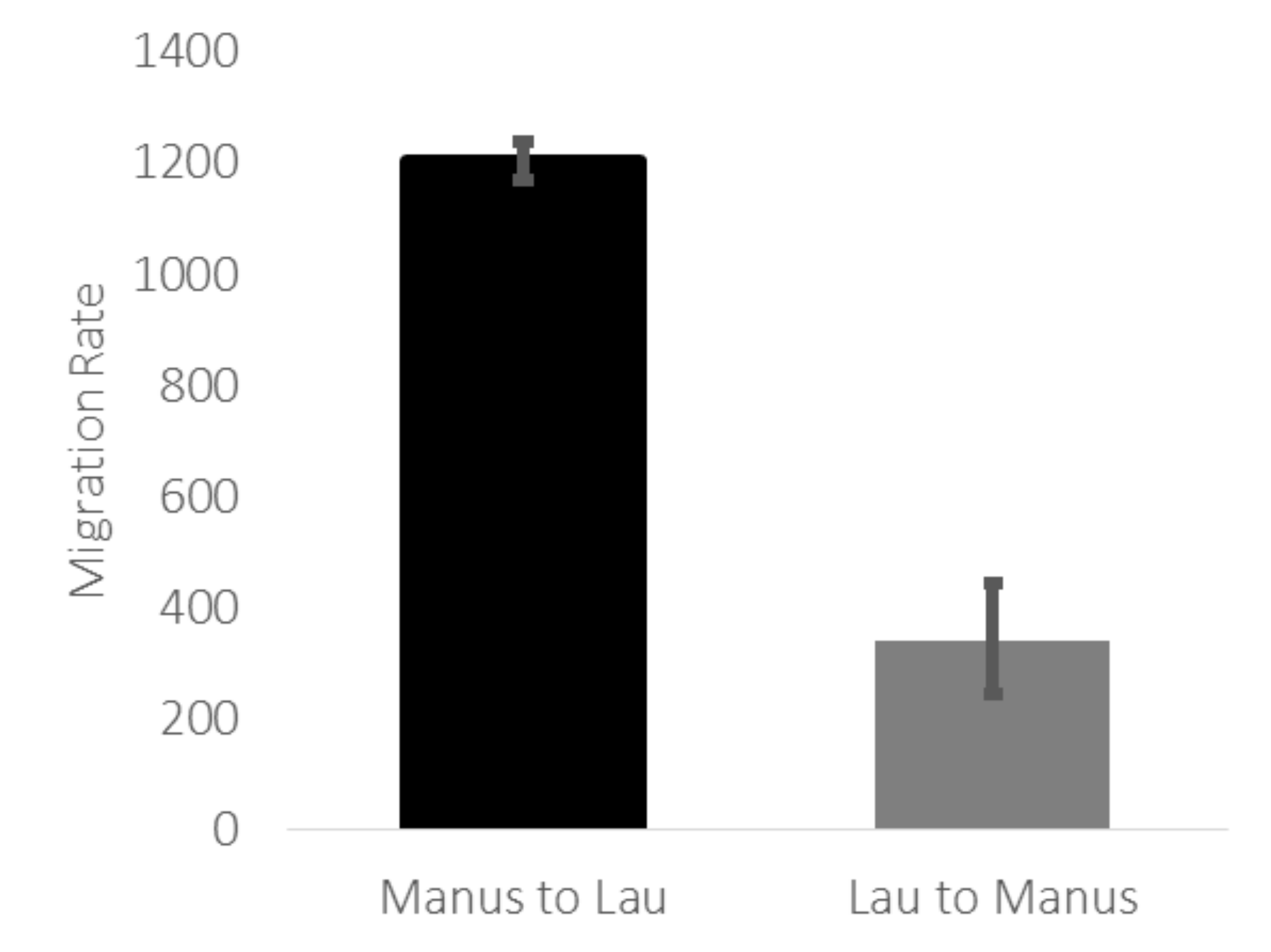

1400
1200
1000
800
600
400
200
0
Migration Rate
Manus to Lau
Lau to Manus
